# Twenty common errors in the diagnosis and treatment of periprosthetic joint infection

**DOI:** 10.1007/s00264-019-04426-7

**Published:** 2019-10-22

**Authors:** Cheng Li, Nora Renz, Andrej Trampuz, Cristina Ojeda-Thies

**Affiliations:** 1 Charité – Universitätsmedizin Berlin, corporate member of Freie Universität Berlin, Humboldt-Universität zu Berlin, and Berlin Institute of Health, Center for Musculoskeletal Surgery (CMSC), Charitéplatz 1, 10117 Berlin, Germany; 2 Department of Traumatology and Orthopedic Surgery, Hospital Universitario 12 de Octubre, Madrid, Spain

**Keywords:** Periprosthetic joint infection, Synovial fluid analysis, Hip arthroplasty, Knee arthroplasty, Joint replacement surgery

## Abstract

**Background:**

Misconceptions and errors in the management of periprosthetic joint infection (PJI) can compromise the treatment success. The goal of this paper is to systematically describe twenty common mistakes in the diagnosis and management of PJI, to help surgeons avoid these pitfalls.

**Materials and methods:**

Common diagnostic and treatment errors are described, analyzed and interpreted.

**Results:**

Diagnostic errors include the use of serum inflammatory biomarkers (such as C-reactive protein) to rule out PJI, incomplete evaluation of joint aspirate, and suboptimal microbiological procedures (such as using swabs or collection of insufficient number of periprosthetic samples). Further errors are missing possible sources of distant infection in hematogenous PJI or overreliance on suboptimal diagnostic criteria which can hinder or delay the diagnosis of PJI or mislabel infections as aseptic failure. Insufficient surgical treatment or inadequate antibiotic treatment are further reasons for treatment failure and emergence of antimicrobial resistance. Finally, wrong surgical indication, both underdebridement and overdebridement or failure to individualize treatment can jeopardize surgical results.

**Conclusion:**

Multidisciplinary teamwork with infectious disease specialists and microbiologists in collaboration with orthopedic surgeons have a synergistic effect on the management of PJI. An awareness of the possible pitfalls can improve diagnosis and treatment results.

## Introduction

Prosthetic joint infection (PJI) is a serious complication of joint replacement surgery, requiring extended periods of hospitalization and re-operations and posing a significant financial burden. PJI has been found to be the most common cause of failure after hip arthroplasty [1] and of early failure after knee replacement [2]. In spite of increased interest and advances in the diagnosis and management of PJI [3–7], it remains a challenge for the treating physicians, and recent meta-analyses have reported microbiological failure rates of 0–40% for one- and two-stage revision for infected hip and knee arthroplasties [8–12]. Failure can be due to patient factors, microbiological factors [13], or factors related to errors during diagnosis and treatment [14, 15]. The goal of this article is to provide a summary of the possible pitfalls and errors during the process of diagnosing and treating PJI.

## Diagnostic errors in PJI

### Delayed diagnosis

Infection should be ruled out in any patient with persistent wound leakage, or a warm, swollen, or painful joint. Unfortunately, it is common to minimize the problem, taking a “wait and see” approach, losing valuable time, as the effectiveness of debridement, antibiotics, and implant retention (DAIR) decreases as surgical delay increases [16]. Bacteria adhere on implants within seconds and start surrounding themselves by a self-produced extracellular polysaccharide matrix; mature biofilm with the more difficult-to-eradicate sessile bacteria can be found at approximately three weeks [17, 18].

### Use of swab samples

Microbiological culture samples from tissue swabs should be avoided. Previous reports have shown that the sensitivity of swab culture is low (53–76%) and is often associated with misidentification of causative pathogens [19]. Results of swab-based samples from draining wounds or sinus tracts are misleading, as they are likely to produce polymicrobial or false-positive results due to contamination with skin flora such as coagulase-negative staphylococci and *Cutibacterium* spp. The concordance with deep tissue samples is low (53%) [20].

### Use of serum C-reactive protein and erythrocyte sedimentation rate to rule out infection

Surgeons frequently report using C-reactive protein (CRP) and erythrocyte sedimentation rates (ESRs) as first-line tests in suspected PJI, because of their convenience and short waiting times. They are strongly recommended in the 2010 guidelines of the American Association of Orthopaedic Surgeons (AAOS) [21, 22]. However, CRP and ESR are inflammatory markers with a low sensitivity: levels within the normal range do not rule out infection. Pérez-Prieto et al. [23] found that one third of PJI presented normal CRP levels and that approximately two thirds of these also had a normal ESR, and Akgün et al. [24] had similar findings. This is the case especially in low-grade infections due to coagulase-negative staphylococci and *Cutibacterium* spp. [25] or in patients under antibiotic treatment [26]. Though elevated ESR and/or CRP levels were included as a diagnostic criterion in the 2011 definition of PJI by the Musculoskeletal Infection Society (MSIS) [27] and the 2013 guidelines of the Infectious Diseases Society of America (IDSA) [4], the 2018 Proceedings of International Consensus Meeting (ICM) on Orthopedic Infections underlined that negative test results do not exclude the possibility of infection [28].

### Disregarding distant sources of infection

**Fig. 1:**
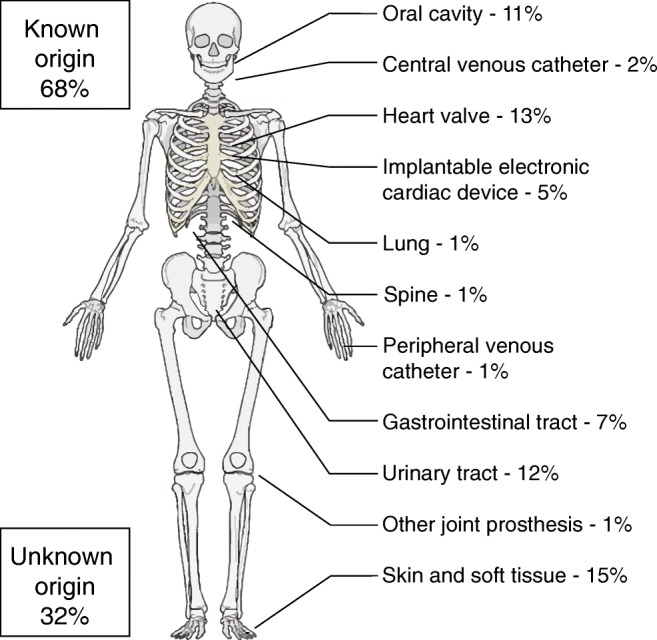
Origin of haematogenous infection (adapted from Rakow et al. [29])

Haematogenous spread from a distant infectious focus onto the prosthesis through filtration of bacteria during bacteremia is the second most common pathogenesis after peri-operative contamination. Persistent sources of infection should be considered if inflammatory biomarker levels do not fall steadily after initiating PJI treatment or when symptoms present acutely after a prolonged pain-free period following initial implantation. Primary infectious foci can be identified in the majority of acute haematogenous infections [29]. Common sources are the cardiovascular system, skin and soft tissue, oral cavity, and urogenital and gastrointestinal tracts (Fig. 1).

### Incomplete synovial fluid analysis

Arthrocentesis is the most commonly performed pre-operative invasive test for suspected PJI. However, in a survey among European surgeons, many focused merely on synovial fluid culture, with one in four ignoring the diagnostic value of aspirate leukocyte count and polymorphonuclear cell (PMN) percentage [21]. However, synovial fluid culture has been found to have a relatively low sensitivity and specificity [30], and over reliance on identifying a micro-organism pre-operatively in the synovial fluid can miss cases of PJI. In addition, knowing the causal micro-organism pre-operatively—or not—does not compromise reinfection rates after treatment [31]. A recent meta-analysis of ten studies found that synovial fluid white blood cell (WBC) count had a sensitivity of 90.0% (95% confidence interval (CI) 87.2–92.2%) and a specificity of 89.8% (95% CI 81.4–94.7%), though with differences when regarding total hip and total knee arthroplasties and when considering different thresholds [32, 33]. The threshold of ≥ 1700 WBC/mm^3^ recommended in the 2010 AAOS guidelines [22] and the 2013 IDSA guidelines [4] had a higher sensitivity but lower specificity than the ≥ 3000 WBC/mm^3^ suggested in the 2018 ICM Proceedings [28]. Synovial WBC count and PMN percentages are unable to detect pathogens but can help discern true-positive and false-positive results. Furthermore, PMN percentage is not affected by antibiotic treatment [34]. Synovial fluid cell count may, however, be increased in patients with rheumatic arthritis, in patients with periprosthetic fractures, and in the early post-operative period following joint replacement, with false-positive results more likely in these settings [34, 35]. Other diagnostic tests have been popularized in the last decade such as alpha defensin. Predominantly in low-grade infections, the sensitivity of this biomarker and synovial polymerase chain reaction (PCR), but their diagnostic performance has been found to be inferior to conventional diagnostic methods, with several authors recommending their use as a confirmatory tool in equivocal cases rather than as a screening tool [35, 36]. Molecular tests such as synovial PCR did not outperform conventional culture in general, except in infections caused by low-virulent pathogens [37, 38]. The clinical value of next generation sequencing (NGS) in the diagnosis of culture-negative PJI is currently investigated.

### Misinterpretation of macroscopic purulence as infection in the presence of metal-on-metal bearings

Alijanipour et al. [39] found that the presence of purulence was poorly associated with isolation of a micro-organism from culture; its diagnostic accuracy was 77%. However, several guidelines include the presence of purulence in the joint as a definite criterion for the diagnosis of periprosthetic joint infection, providing that metal-on metal (MoM) bearings were excluded [3, 4, 27]. In MoM bearings, hypersensitivity reaction and aseptic inflammation can also generate purulent appearance (“pseudo-purulence”), along with joint pain, increased serum CRP, and other symptoms of suspected infection [40]. The fluid and tissue analyses in MoM bearings show highly increased levels of cobalt and chromium [41], including in patients with “trunniasis,” where corrosion and debris occur in the taper-stem junction of modular hip prostheses [42].

### Inadequate number of periprosthetic tissue samples for bacterial culture

**Fig. 2:**
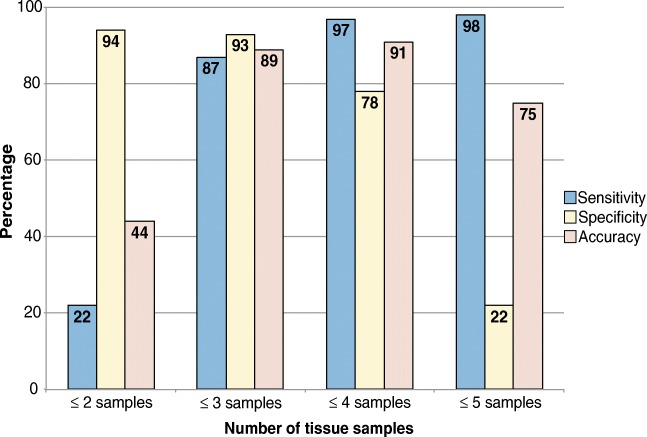
Sensitivity, specificity, and accuracy of diagnosis of PJI with two or more samples using conventional culture methods (using Bayesian latent class modeling) (extracted from the data in Peel et al. [43])

The most commonly used intra-operative diagnostic method is tissue sampling for culture. The IDSA recommends submitting at least three and optimally five or six periprosthetic intra-operative tissue samples for aerobic and anaerobic culture [4]. Peel et al. [43] found that five or more tissue samples did not improve diagnostic accuracy, and recommended using three samples of periprosthetic tissue in blood culture bottles (BCBs) or four samples in conventional culture (Fig. 2).

### Errors during retrieval of diagnostic samples

Several pitfalls during tissue sampling can increase the risk of false-positive or false-negative results. First, tissue samples should be obtained using sharp dissection, avoiding the use of electrocautery in order to limit false-positive results due to thermal artifacts in histopathologic analysis [44, 45]. Second, samples should be retrieved from the areas where signs of infection are more pronounced and from different areas of the surgical field (e.g., in hip revisions, from the bottom of the acetabulum and from the femoral canal) [15]. Third, surgical instruments should be changed for each tissue sample to avoid a risk of cross-contamination between samples, which could impact culture results [46]. Fourth, sonication of the removed implants in polyethylene bags increases the risk of microbial contamination leading to a false-positive result [47]. Finally, when transferring synovial fluid into an EDTA tube, thorough immediate hand mixing is necessary to avoid coagulation of the sample, as this would influence the synovial WBC [48].

### Overdependence on diagnostic criteria

**Fig. 3:**
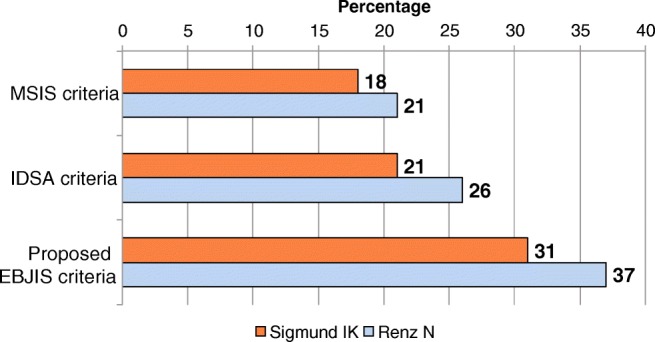
Percentage of patients diagnosed with periprosthetic joint infection using the diagnostic criteria proposed by different scientific societies (Musculoskeletal Infection Society (MSIS) criteria, IDSA criteria, and the proposed European Bone and Joint Infection Society (EBJIS, working draft) criteria. Data extracted from references [35, 49]

All guidelines on PJI should be considered auxiliary tools for physicians diagnosing infection; a few cases of PJI may be missed, or cases of aseptic loosening may be mistakenly diagnosed as PJI, as the sensitivity and specificity of the diagnostic criteria proposed by scientific societies do not reach 100%, and the percentage of patients diagnosed with PJI within a patient cohort varies considerably depending on the diagnostic criteria used (Fig. 3) [35, 49, 50].

## Treatment errors in PJI

### Conservative treatment with antibiotics in early infections

Antibiotic treatment is commonly initiated in cases of fever, chills, persistent inflammation, or wound drainage following joint arthroplasty surgery. It is difficult to differentiate superficial wound infection from early post-operative prosthetic joint infection, and wound issues are a risk factor for PJI [51, 52]. Long-term antibiotic therapy with the intent of limiting inflammation and improving clinical symptoms without prior revision surgery has not been found to eradicate infection. As stated above, delayed surgery compromises the likelihood of success of treatment. Prompt surgery followed by antibiotic therapy is the cornerstone of successful treatment of PJI. Suppressive antibiotic therapy has limited clinical efficacy and is associated with a substantial risk of adverse effects and should be reserved for patients in whom further surgical treatment is unadvisable (i.e., extreme frailty, comorbidities) [53].

### Antibiotic treatment prior to microbiological diagnosis

Corollary of the aforementioned point, the diagnostic accuracy of synovial fluid culture results is compromised if synovial fluid aspiration is performed after initiating antibiotic therapy, increasing the risk of false-negative results. The same is true for peri-operative tissue cultures, and preoperative antibiotic therapy is the most important cause of culture-negative PJI [52]. If diagnostic measures are planned in a patient currently being under antimicrobial treatment, antibiotics should be withheld for two weeks prior to microbiological sampling. Nevertheless, culture of sonicate fluid could improve the diagnostic accuracy of patients under antimicrobial treatment [47, 54].

### Failure to individualize treatment

Treatment of an infected prosthesis should be tailored to the type of infection (early/acute or late/chronic infection), the causative micro-organism, the quality of the soft tissue envelope, stability of the implant, surgeon experience, and ultimately host factors (comorbidities and functional status) and patient preferences [55, 56]. In acute PJI, the duration of symptoms is less than three weeks (haematogenous or contiguous infections) or four weeks (early post-operative infections); all other infections with longer duration of symptoms are defined as chronic PJI (Table 1) [6]. Implant retention using the DAIR strategy can be used in acute PJI; all mobile parts should, however, be exchanged, as patients in whom all modular components have been exchanged are shown to have higher treatment success rates [16, 57, 58]. Other requisites for success with DAIR are sufficient debridement, infection due microorganisms sensitive to biofilm-active antibiotics, a stable arthroplasty, and good soft tissue envelope. Chronic PJI presents with a mature biofilm; thus, the prosthesis must be exchanged. This can be performed in one or two stages, depending on the causative micro-organisms, soft tissue condition, and surgeon and patient preference. While two-stage exchange of an infected prosthesis is considered the gold standard and is the dominant option in the USA, single-stage exchange is favoured in some European countries. One-stage exchange may not be an option in patients with signs of systemic sepsis, extensive comorbidities, infection with resistant organisms, culture-negative infections, and poor soft tissue coverage [59–61]. With proper selection of surgery, success rates of PJI treatment can exceed 80–90% [59].

**Table 1:** Classification, characteristics, and treatment strategies of PJI

	Acute PJI (immature biofilm)	Chronic PJI (mature biofilm)
Pathogenesis		
- Peri-operative	< 4 weeks after surgery	≥ 4 weeks after surgery (typically 3 months–3 years)
- Haematogenous or contiguous	< 3 weeks of symptoms	≥ 3 weeks of symptoms
Clinical features	*Acute pain*, fever, red/swollen joint, prolonged postoperative discharge (> 7–10 days)	*Chronic pain*, loosening of the prosthesis, sinus tract (fistula)
Causative micro-organism	*High-virulent*: *Staphylococcus aureus*, gram-negative bacteria (e.g., *Escherichia coli*, *Klebsiella*, *Pseudomonas aeruginosa*)	*Low-virulent*: coagulase-negative staphylococci (e.g., *Staphylococcus epidermidis*), *Cutibacterium acnes*
Surgical treatment	*Debridement and retention of prosthesis* (change of mobile parts)	*Complete removal of prosthesis* (exchange in one or two stages)

Adapted from Li et al. [6]

In patients too frail or too sick for surgery that have low functional demand or who reject surgical treatment, improvement of the patient’s quality of life should be the goal of treatment, with or without the use of antibiotics. The success rate of suppressive antimicrobial therapy has been reported to be between 23 and 83% [53, 62]. In a series of six patients in stable condition and with well-fixed prostheses treated expectantly withholding antibiotic therapy, Giacometti Ceroni et al. [63] reported that 83.3% of patients were pain-free and without systemic symptoms after a mean follow-up of 6.7 years (range 2–10 years).

### Arthroscopic lavage for treatment of PJI

Arthroscopic lavage of an infected prosthetic joint does not allow access to all parts of the joint, particularly the posterior part of the knee joint and the polyethylene liner backside in knee as well as other joints. In addition, it does not allow for exchange of mobile parts, reflecting insufficient debridement as mentioned above (error 12). In prosthetic hip joints, debridement is insufficient without dislocation of the femoral head, which is difficult to perform without arthrotomy. Byren et al. [64] observed four times higher failure rate following arthroscopic lavage than standard DAIR surgery. Hyman et al. [65] described favorable results in a series of eight patients with late acute hip PJIs—all patients were, however, managed with chronic antibiotic suppression in addition to arthroscopic lavage. Arthroscopy has a limited role in the diagnostic workup of a painful prosthesis, allowing for inspection of the components in search of instability and wear, exclusion of non-infectious causes, visualization of the synovium, and retrieval of samples for microbiology and histology in selected cases [14, 66–68]. Importantly, arthroscopy is an invasive intervention, which is associated with a small risk of infection; therefore, the indication for diagnostic arthroscopy should be considered carefully.

### Insufficient debridement or incomplete exchange of implants

A common reason for treatment failure is inadequate debridement. All diseased or devitalized tissue and bone should be removed during surgery. This includes old scar tissue, sinus tracts, osteolytic regions, sequestra, and any devitalized tissue until bleeding margins are obtained. In infections with mature biofilm, all foreign material including cerclages and bone cement should be rigorously removed. Although some series have documented partial exchange of implants with acceptable results, particularly in cases in which a prosthetic component is so well-fixed that its removal could result in significant bone loss and compromise of fixation at the time of the later prosthesis reimplantation and the causative organisms are not multidrug-resistant, in immunocompetent patients without sinus tracts, this option should be the exception rather than the norm [15, 66, 69, 70], and surgeons should be aware that this could compromise treatment success.

### High-pressure pulse lavage during surgery

Pulse lavage is commonly used in PJI surgery. In the clinical setting, the success rate in treating orthopaedic implant–related infections is similar when using high-pressure and low-pressure pulsatile lavages (81.6% vs. 84.4%, respectively; *p* = 1.00) [71]. Several in vitro studies have shown, however, that pulse lavage may not be suitable for PJI surgery, especially in cases of DAIR. Not only is pulse lavage ineffective in removing biofilms from the implant surface [72], it can also potentially increase soft tissue damage and propagate bacteria deeper into soft tissue, leading to increased bacterial retention [73].

### Errors using antibiotic-loaded cement spacers

The use of antibiotic-loaded bone cement (ALBC) spacers in two-stage revisions has two goals: first, it provides local delivery of high doses of antibiotics, above the minimal inhibitory concentration (MIC) normally attainable locally with systemic treatment without adverse effects. Second, it serves as a filler of dead space, limiting the presence of void-filling haematoma, avoiding joint contractures, and increasing joint stability and even mobility [74]. The drug chosen for the mixture has to meet the following requirements: (1) it needs to be thermostable enough to still be effective after the exothermic reaction of polymethyl methacrylate (PMMA) polymerization, (2) it should not interfere with the polymerization process (i.e., impede hardening of PMMA), (3) it has to be able to elute from the bone cement after hardening, (4) it has to be hydrosoluble to diffuse into the surrounding tissues, and (5) it has to be available in powder form, as adding liquid antibiotics to the cement mixture significantly decreases its mechanical strength. Many premixed ALBC mixtures are commercially available; most, however, have relatively low doses of antibiotics and are primarily intended for fixation use in reimplantation surgery in two-stage exchanges or for primary arthroplasties in high-risk patients. Furthermore, hand-mixed ALBC allows for individual tailoring of the spacer to the causative micro-organism (Table 2). Common pitfalls are using antibiotics inadequate for mixing with bone cement or that are not effective for the type of micro-organism treated (e.g., vancomycin for gram-negative bacteria). Antibiotics elute from ALBC in a negatively exponential fashion, with high doses in the first post-operative days and a loss of therapeutic levels after days to weeks, depending on the type and dose of antibiotic and cement used. Thus, the risk of the spacer acting as a foreign body at risk for biofilm formation increases as time passes. Tan et al. [75] found that the risk of reinfection increased as the spacer was left implanted for longer periods, with a clear inflection above 100 days.

**Table 2:** Dosage of antibiotics mixed in antibiotic-loaded bone cement (ALBC)

Situation	Antimicrobial	Fixation cement (prophylactic dose: per 40 g PMMA cement)	Spacer cement (therapeutic dose: per 40 g PMMA cement)
		Simple: industrially admixed antibiotics(italics: manually admixed antibiotics)	
Standard situation			
Susceptible or unknown pathogen(s)	Gentamicin +	1 g	1 g
	Clindamycin	1 g	1 g (*+ 2 g vancomycin*)
Special situations			
*Staphylococcus* spp. (oxacillin-/methicillin-resistant)	Gentamicin +	0.5 g	0.5 g
	Vancomycin or	2 g	2 g (*+ 2 g*^a^)
	*Daptomycin*	*2 g*	*3 g*
Vancomycin-resistant enterococci (VRE)	Gentamicin +	0.5 g	0.5–1 g
	*Linezolid* or	*1 g*	*2 g*
	*Daptomycin* or	*2 g*	*3 g*
	*Fosfomycin sodium*^b^	*2 g*	*2-4 g*
Resistant gram-negative pathogens (e.g., *E. coli*, *Klebsiella*, *Enterobacter*, *Pseudomonas* spp.)	Gentamicin +	0.5 g	0.5–1 g
	*Colistin*^c^ or	*2 g* (*= 60 million U*)	*4 g* (*= 120 million U*)
	*Fosfomycin sodium*^b^ or	*2 g*	*2-4 g*
	*Meropenem* or	*2 g*	*3 g*^d^
	*Ciprofloxacin*	*2 g*	*3 g*
Yeasts (*Candida* spp.) or molds (e.g., *Aspergillus* spp.)	Gentamicin +	0.5 g	0.5–1 g
	*Liposomal amphotericin B (Ambisome®)* or	*0.2 g*^e^	*0.2 g*^a, e^
	*Voriconazole*	*0.2 g*	*0.4 g*^a^

^a^These Atb concentrations do not fulfill the mechanical ISO requirements for fixation cement

^b^Fosfomycin sodium is preferred over fosfomycin calcium due to better mechanical properties of PMMA

^c^Available as colistin sodium or colistin sulfate (equal efficacy)

^d^Improved efficacy and antimicrobial release in combination with gentamicin 1 g and clindamycin 1 g

^e^The literature is still controversial regarding minimal effective concentrations

### “Antibiotic holiday” and joint aspiration before re-implantation in two-stage exchange surgery

**Fig. 4:**
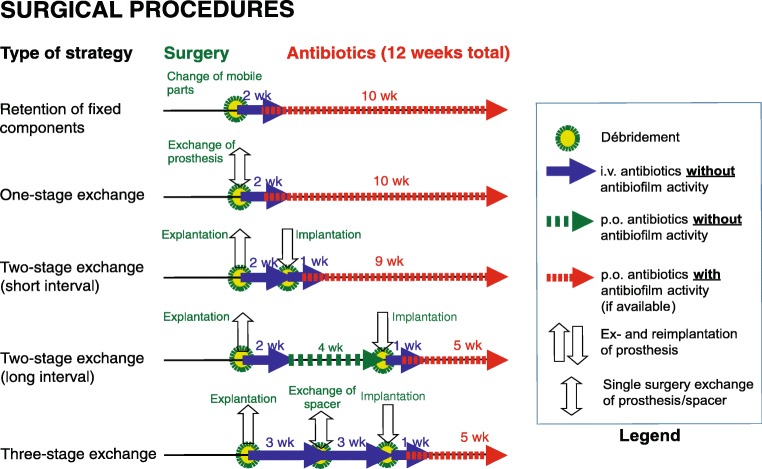
Algorithms of the different treatment modalities of PJI. Adapted from Li et al. [6]

Currently, there is insufficient evidence to support ceasing antibiotic treatment before reimplantation to confirm eradication of infection (Fig. 4). The duration of an antibiotic holiday seems to be less related with reinfection than the time the antibiotic cement spacer is implanted [75]. Joint aspiration before reimplantation is not recommended, as synovial markers do not correlate with reinfection rates, and the diagnostic accuracy is very low (sensitivity of 4.6% and 25.0% and specificity of 94.3% and 96.9% for synovial fluid and cell count, respectively) [75–77]. To date, there is no reliable marker to prove the eradication of infection at the time of reimplantation. In a recent study, the outcome of PJI treatment remained high despite omission of the antibiotic-free interval before re-implantation [78].

### Errors in the selection of antibiotic treatment

Antibiotic treatment should be based on the type of microorganism, drug susceptibility, and the type of surgery performed (Table 3). Not all antibiotics are equally active against sessile bacteria embedded in biofilm (examples of biofilm active antibiotics are rifampicin for several gram-positive pathogens (e.g., *Staphylococcus* species, *Cutibacterium* species) and ciprofloxacin for gram-negative rods), and these should be reserved for the period after implantation of the definitive implant (Fig. 4). In two-stage exchanges, we do not recommend using the antibiotics in the prosthesis-free interval but rather initiate them once the prosthesis has been re-implanted. In one-stage exchanges and infections treated with DAIR, biofilm-active therapy should be initiated post-operatively as soon as the wounds are dry and drains removed [6]. Another error is prescribing oral antibiotics with bad bone penetration and poor oral bioavailability, resulting in insufficient local concentrations at the site of infection (e.g., beta-lactam antibiotics). Furthermore, single-drug regimens such as rifampin monotherapy should be avoided in order to minimize the risk of selecting drug-resistant micro-organisms [59].

**Table 3:** Targeted antibiotic therapy regimens

Micro-organism (bold-italics: difficult-to-treat)	Antibiotic^a^ (check pathogen susceptibility before)	Dose^b^ (italics: renal adjustment needed)	Route
*Staphylococcus* spp.			
Oxacillin-/methicillin-susceptible	Flucloxacillin^c^	*4 × 2 g*	i.v.
	(± Fosfomycin)	(*3 × 5 g*)	i.v.
	For 2 weeks, followed by (according to susceptibility)		
	Rifampicin^d^ +	2 × 450 mg	p.o.
	Levofloxacin or	*2 × 500 mg*	p.o.
	Cotrimoxazole or	*3 × 960 mg*	p.o.
	Doxycyclin or	2 × 100 mg	p.o.
	Fusidic acid	3 × 500 mg	p.o.
Oxacillin-/methicillin-resistant	Daptomycin or	*1 × 8 mg/kg*	i.v.
	Vancomycin^e^	*2 × 1 g*	i.v.
	(± Fosfomycin)	(*3 × 5 g*)	i.v.
	For 2 weeks, followed by an oral *rifampin* combination as above		
***Rifampicin-resistant***	Intravenous treatment according susceptibility for 2 weeks (as above), followed by long-term suppression for ≥ 1 year		
*Streptococcus* spp.	Penicillin G^c^ or	*4 × 5 million U*	i.v.
	Ceftriaxone	*1 × 2 g*	i.v.
	For 2–4 weeks, followed by		
	Amoxicillin or	*3 × 1000 mg*	p.o.
	Doxycycline	*2 × 100 mg*	p.o.
	(Consider suppression for 1 year)		
*Enterococcus* spp.			
Penicillin-susceptible	Ampicillin +	*4 × 2 g*	i.v.
	Gentamicin^f^	*1 × 120 mg*	i.v.
	(± Fosfomycin)	(*3 × 5 g*)	i.v.
	For 2–3 weeks, followed by		
	Amoxicillin	*3 × 1000 mg*	p.o.
***Penicillin-resistant***	Vancomycin^e^ or	*2 × 1 g*	i.v.
	Daptomycin +	*1 × 10 mg/kg*	i.v.
	Gentamicin^f^	*1 × 120 mg*	i.v.
	(± Fosfomycin)	(*3 × 5 g*)	i.v.
	For 2–4 weeks, followed by		
	Linezolid (max. 4 weeks)	2 × 600 mg	p.o.
Vancomycin-resistant (VRE)	Individual; removal of the implant or lifelong suppression necessary, e.g., with doxycycline (if susceptible)		
Gram-negative			
Enterobacteriaceae (*E. coli*, *Klebsiella*, *Enterobacter*, etc.)	Ciprofloxacin^g^	*2 × 750 mg*	p.o.
Non-fermenters (*Pseudomonas aeruginosa*, *Acinetobacter* spp.)	Piperacillin/tazobactam or	*3 × 4.5 g*	i.v.
	Meropenem or	*3 × 1 g*	i.v.
	Ceftazidime +	*3 × 2 g*	i.v.
	Tobramycin	*1 × 300 mg*	i.v.
	(or gentamicin)	*1 × 240 mg*	i.v.
	For 2–3 weeks, followed by		
	Ciprofloxacin	*2 × 750 mg*	p.o.
***Ciprofloxacin-resistant***	Depending on susceptibility: *meropenem 3 × 1 g*, *colistin 3 × 3 million U*, and/or *fosfomycin 3 × 5 g i.v.*, followed by oral suppression		
Anaerobes			
Gram-positive (*Cutibacterium*, *Peptostreptococcus*, *Finegoldia magna*)	Penicillin G^c^ or	*4 × 5 million U*	i.v.
	Ceftriaxone	*1 × 2 g*	i.v.
	For 2 weeks, followed by		
	Rifampin^d^ +	2 × 450 mg	p.o.
	Levofloxacin or	*2 × 500 mg*	p.o.
	Amoxicillin	*3 × 1000 mg*	p.o.
Gram-negative (*Bacteroides* or *Fusobacterium* spp.)	Ampicillin/sulbactam^c^	*3 × 3 g*	i.v.
	For 2 weeks, followed by		
	Metronidazole	3 × 400 mg or 3 × 500 mg	p.o.
*Candida* spp.			
***Fluconazole-susceptible***	Caspofungin^h^	1 × 70 mg	i.v.
	Anidulafungin	1 × 100 mg (1st day, 200 mg)	i.v.
	For 1–2 weeks, followed by		
	Fluconazole (suppression for ≥ 1 year)	*1 × 400 mg*	p.o.
***Fluconazole-resistant***	Individual (e.g., with voriconazole 2 × 200 mg p.o.); removal of the implant or long-term suppression		
Culture-negative	Ampicillin/sulbactam^c^	*3 × 3 g*	i.v.
	For 2 weeks, followed by		
	Rifampin^d^ +	2 × 450 mg	p.o.
	Levofloxacin	*2 × 500 mg*	p.o.

^a^The total duration of therapy is 12 weeks, usually 2 weeks intravenously, followed by oral route

^b^Laboratory testing 2*×* weekly: leukocytes, CRP, creatinine/eGFR, and liver enzymes (AST/SGOT and ALT/SGPT); dose adjustment according to *renal function* and body weight (< 40/> 100 kg)

^c^Penicillin allergy of non-type 1 (e.g., skin rash): cefazolin (3 × 2 g i.v.); in case of anaphylaxis (= type 1 allergy such as Quincke’s edema, bronchospasm, anaphylactic shock) or cephalosporin allergy: vancomycin (2 × 1 g i.v.) or daptomycin (1 × 8 mg/kg i.v.). Ampicillin/sulbactam is equivalent to amoxicillin/clavulanic acid (3 × 2.2 g i.v.)

^d^Rifampin is administered only after the new prosthesis is implanted. Add it already to intravenous treatment as soon as wounds are dry and drains removed; in patients aged > 75 years, rifampin is reduced to 2 × 300 mg p.o.

^e^Check vancomycin through concentration (take blood before next dose) at least 1×/week; therapeutic range 15–20 μg/ml

^f^Give only, if gentamicin high level (HL) is tested susceptible (consult the microbiologist). In gentamicin HL-resistant *Enterococcus faecalis*, gentamicin is exchanged with ceftriaxone (1 × 2 g i.v.)

^g^Add i.v. treatment (piperacillin/tazobactam 3 × 4.5 g or ceftriaxone 1 × 2 g or meropenem 3 × 1 g i.v.) in the first postoperative days (until wound is dry)

^h^After a loading dose of 70 mg on day 1, reduce dose to 50 mg in patients weighing < 80 kg from day 2

### Inadequate management of soft tissues

Occasionally, there are difficulties for adequate closure of the soft tissue envelope surrounding a joint arthroplasty. A plastic surgeon should be consulted promptly, because any exposed prosthesis will quickly be colonized with micro-organisms and covered in biofilm. Wound revision, with revision of the prosthesis if necessary, is always indicated, as it is the only way to guarantee a favourable course, both from the infectious and from the functional point of view. Negative pressure wound therapy should not be used, as it can lead to superinfection with gram-negative or multiresistant micro-organisms [79]; if inevitable, its use should be limited in time, only as a short bridging therapy of a few days before the plastic surgeon can perform definite coverage with local or free flaps [14, 15, 55].

### Lack of specialized multidisciplinary teamwork

The diagnosis and treatment of PJI involves multiple steps, including but not limited to the evaluation of the patient, particularly the affected joint, the interpretation of synovial fluid analysis and bacterial culture results, the development and execution of the surgical plan, and the choice of antibiotic treatment. This involves at least microbiologists, infectious disease specialists, and orthopaedic and plastic surgeons. The orthopedic surgeon should not choose the antibiotic cocktail alone, as choosing the right regimen is difficult. It is essential that the surgeon works very closely with an infectious disease specialist experienced in the treatment of implant infections. Conversely, the infectious disease specialist should not propose surgical treatments without understanding their impact and their importance to the patient and without knowing the different techniques employed by the surgeon. A microbiologist can assist in both the correct retrieval of samples and the processing of these in order to provide an exact diagnosis. Though, to our knowledge, no study has evaluated multidisciplinary interventions in a randomized manner [80], several authors have shown that a multidisciplinary protocol provides excellent results, with a lower length of in-hospital stay, number of surgeries, and number of antibiotics required [14, 31, 55, 81–84].

## Conclusions

Management of prosthetic joint infections is a challenge, and there are many possible sources of errors in the diagnosis and treatment of these patients. A thorough understanding of the biology of these infections and the advantages and limitations of existing diagnostic tests and surgical options is essential. Awareness of the potential pitfalls and a systematic approach can improve the likelihood of success.

## References

[1] Jafari SM, Coyle C, Mortazavi SMJ (2010). Revision hip arthroplasty: infection is the most common cause of failure. Clin Orthop Relat Res.

[2] Thiele K, Perka C, Matziolis G (2015). Current failure mechanisms after knee arthroplasty have changed: polyethylene wear is less common in revision surgery. J Bone Joint Surg Am.

[3] Parvizi J, Gehrke T, Mont MA, Callaghan JJ (2019). Introduction: proceedings of International Consensus on Orthopedic Infections. J Arthroplast.

[4] Osmon DR, Berbari EF, Berendt AR (2013). Diagnosis and management of prosthetic joint infection: clinical practice guidelines by the Infectious Diseases Society of America. Clin Infect Dis.

[5] Arvieux C, Common H (2019). New diagnostic tools for prosthetic joint infection. Orthop Traumatol Surg Res.

[6] Li C, Renz N, Trampuz A (2018). Management of periprosthetic joint infection. Hip Pelvis.

[7] Li C, Renz N, Thies CO, Trampuz A (2018). Meta-analysis of sonicate fluid in blood culture bottles for diagnosing periprosthetic joint infection. J Bone Jt Infect.

[8] Leonard HAC, Liddle AD, Burke O (2014). Single- or two-stage revision for infected total hip arthroplasty? A systematic review of the literature. Clin Orthop Relat Res.

[9] Masters JPM, Smith NA, Foguet P (2013). A systematic review of the evidence for single stage and two stage revision of infected knee replacement. BMC Musculoskelet Disord.

[10] Nagra NS, Hamilton TW, Ganatra S (2016). One-stage versus two-stage exchange arthroplasty for infected total knee arthroplasty: a systematic review. Knee Surg Sports Traumatol Arthrosc.

[11] Yaghmour Khaled, Chisari Emanuele, Khan Wasim (2019). Single-Stage Revision Surgery in Infected Total Knee Arthroplasty: A PRISMA Systematic Review. Journal of Clinical Medicine.

[12] Kunutsor Setor K., Whitehouse Michael R., Lenguerrand Erik, Blom Ashley W., Beswick Andrew D. (2016). Re-Infection Outcomes Following One- And Two-Stage Surgical Revision of Infected Knee Prosthesis: A Systematic Review and Meta-Analysis. PLOS ONE.

[13] Fagotti L, Tatka J, Salles MJC, Queiroz MC (2018). Risk factors and treatment options for failure of a two-stage exchange. Curr Rev Musculoskelet Med.

[14] Borens O, Tissot C, Delaloye J-R, Trampuz A (2012). Ten errors to avoid while dealing with infected total joint arthroplasties. Rev Med Suisse.

[15] Kocjančič B, Dolinar D (2014). The most common surgical errors in the treatment of prosthetic joint infections. Zdravniški Vestnik.

[16] Tsang S-TJ, Ting J, Simpson AHRW, Gaston P (2017). Outcomes following debridement, antibiotics and implant retention in the management of periprosthetic infections of the hip: a review of cohort studies. Bone Joint J.

[17] Gbejuade HO, Lovering AM, Webb JC (2015). The role of microbial biofilms in prosthetic joint infections. Acta Orthop.

[18] Zimmerli W, Sendi P (2017). Orthopaedic biofilm infections. APMIS.

[19] Aggarwal VK, Higuera C, Deirmengian G (2013). Swab cultures are not as effective as tissue cultures for diagnosis of periprosthetic joint infection. Clin Orthop Relat Res.

[20] Tetreault MW, Wetters NG, Aggarwal VK (2013). Should draining wounds and sinuses associated with hip and knee arthroplasties be cultured?. J Arthroplast.

[21] Ahmad SS, Becker R, Chen AF, Kohl S (2016). EKA survey: diagnosis of prosthetic knee joint infection. Knee Surg Sports Traumatol Arthrosc.

[22] Della Valle C, Parvizi J, Bauer TW (2011). American Academy of Orthopaedic Surgeons clinical practice guideline on: the diagnosis of periprosthetic joint infections of the hip and knee. J Bone Joint Surg Am.

[23] Pérez-Prieto D, Portillo ME, Puig-Verdié L (2017). C-reactive protein may misdiagnose prosthetic joint infections, particularly chronic and low-grade infections. Int Orthop.

[24] Akgün D, Müller M, Perka C, Winkler T (2018). The serum level of C-reactive protein alone cannot be used for the diagnosis of prosthetic joint infections, especially in those caused by organisms of low virulence. Bone Joint J.

[25] Renz N, Mudrovcic S, Perka C, Trampuz A (2018). Orthopedic implant-associated infections caused by Cutibacterium spp.—a remaining diagnostic challenge. PLoS One.

[26] Shahi A, Deirmengian C, Higuera C (2015). Premature therapeutic antimicrobial treatments can compromise the diagnosis of late periprosthetic joint infection. Clin Orthop Relat Res.

[27] Parvizi J, Zmistowski B, Berbari EF (2011). New definition for periprosthetic joint infection: from the Workgroup of the Musculoskeletal Infection Society. Clin Orthop Relat Res.

[28] Bauer TW, Bedair H, Creech JD (2019). Hip and knee section, diagnosis, laboratory tests: proceedings of International Consensus on Orthopedic Infections. J Arthroplast.

[29] Rakow A., Perka C., Trampuz A., Renz N. (2019). Origin and characteristics of haematogenous periprosthetic joint infection. Clinical Microbiology and Infection.

[30] Fernández-Sampedro M, Fariñas-Alvarez C, Garces-Zarzalejo C, et al (2017) Accuracy of different diagnostic tests for early, delayed and late prosthetic joint infection. BMC Infect Dis 17. 10.1186/s12879-017-2693-1 10.1186/s12879-017-2693-1 PMC6389211 28841913

[31] Karczewski Daniel, Winkler Tobias, Perka Carsten, Müller Michael (2018). The Preoperative Microbial Detection is No Prerequisite for the Indication of Septic Revision in Cases of Suspected Periprosthetic Joint Infection. BioMed Research International.

[32] De Fine M, Giavaresi G, Fini M (2018). The role of synovial fluid analysis in the detection of periprosthetic hip and knee infections: a systematic review and meta-analysis. Int Orthop.

[33] Zahar A, Lausmann C, Cavalheiro C (2018). How reliable is the cell count analysis in the diagnosis of prosthetic joint infection?. J Arthroplast.

[34] Trampuz A, Hanssen AD, Osmon DR (2004). Synovial fluid leukocyte count and differential for the diagnosis of prosthetic knee infection. Am J Med.

[35] Renz N, Yermak K, Perka C, Trampuz A (2018). Alpha defensin lateral flow test for diagnosis of periprosthetic joint infection: not a screening but a confirmatory test. J Bone Joint Surg Am.

[36] Kelly MP, Darrith B, Hannon CP (2018). Synovial fluid alpha-defensin is an adjunctive tool in the equivocal diagnosis of periprosthetic joint infection. J Arthroplast.

[37] Suda AJ, Tinelli M, Beisemann ND (2017). Diagnosis of periprosthetic joint infection using alpha-defensin test or multiplex-PCR: ideal diagnostic test still not found. Int Orthop.

[38] Morgenstern C, Cabric S, Perka C (2018). Synovial fluid multiplex PCR is superior to culture for detection of low-virulent pathogens causing periprosthetic joint infection. Diagn Microbiol Infect Dis.

[39] Alijanipour P, Adeli B, Hansen EN (2015). Intraoperative purulence is not reliable for diagnosing periprosthetic joint infection. J Arthroplast.

[40] Biant LC, Bruce WJM, van der Wall H, Walsh WR (2010). Infection or allergy in the painful metal-on-metal total hip arthroplasty?. J Arthroplast.

[41] Rakow A, Schoon J, Dienelt A (2016). Influence of particulate and dissociated metal-on-metal hip endoprosthesis wear on mesenchymal stromal cells in vivo and in vitro. Biomaterials.

[42] Koper MC, Mathijssen NMC, Witt F (2016). Clinical and wear analyses of 9 large metal-on-metal total hip prostheses. PLoS One.

[43] Peel TN, Spelman T, Dylla BL (2017). Optimal periprosthetic tissue specimen number for diagnosis of prosthetic joint infection. J Clin Microbiol.

[44] Zmistowski B, Della Valle C, Bauer TW (2014). Diagnosis of periprosthetic joint infection. J Arthroplast.

[45] Parvizi J, Fassihi SC, Enayatollahi MA (2016). Diagnosis of periprosthetic joint infection following hip and knee arthroplasty. Orthop Clin North Am.

[46] Makki D, Abdalla S, El Gamal T (2018). Is it necessary to change instruments between sampling sites when taking multiple tissue specimens in musculoskeletal infections?. Ann R Coll Surg Engl.

[47] Trampuz A, Piper KE, Hanssen AD (2006). Sonication of explanted prosthetic components in bags for diagnosis of prosthetic joint infection is associated with risk of contamination. J Clin Microbiol.

[48] Ochsner PE, Borens O, Bodler P-M, Schweizerische Gesellschaft für Orthopädie und Traumatologie (2014) Infections of the musculoskeletal system: basic principles, prevention, diagnosis and treatment

[49] Sigmund IK, Yermak K, Perka C (2018). Is the enzyme-linked immunosorbent assay more accurate than the lateral flow alpha defensin test for diagnosing periprosthetic joint infection?. Clin Orthop Relat Res.

[50] Parvizi J, Tan TL, Goswami K (2018). The 2018 definition of periprosthetic hip and knee infection: an evidence-based and validated criteria. J Arthroplast.

[51] Segreti J, Parvizi J, Berbari E (2017). Introduction to the Centers for Disease Control and Prevention and Healthcare Infection Control Practices Advisory Committee guideline for prevention of surgical site infection: prosthetic joint arthroplasty section. Surg Infect.

[52] Johnson DP, Bannister GC (1986). The outcome of infected arthroplasty of the knee. J Bone Joint Surg (Br).

[53] Tsukayama DT, Wicklund B, Gustilo RB (1991). Suppressive antibiotic therapy in chronic prosthetic joint infections. Orthopedics.

[54] Trampuz A, Piper KE, Jacobson MJ (2007). Sonication of removed hip and knee prostheses for diagnosis of infection. N Engl J Med.

[55] Baker RP, Furustrand Tafin U, Borens O (2015). Patient-adapted treatment for prosthetic hip joint infection. Hip Int.

[56] Izakovicova P, Borens O, Trampuz A (2019). Periprosthetic joint infection: current concepts and outlook. EFORT Open Rev.

[57] Zhang C, Yan CH, Chan PK (2017). Polyethylene insert exchange is crucial in debridement for acute periprosthetic infections following total knee arthroplasty. J Knee Surg.

[58] Choi H-R, von Knoch F, Zurakowski D (2011). Can implant retention be recommended for treatment of infected TKA?. Clin Orthop Relat Res.

[59] Zimmerli W (2014). Clinical presentation and treatment of orthopaedic implant-associated infection. J Intern Med.

[60] Renner L, Perka C, Trampuz A, Renz N (2016). Treatment of periprosthetic infections. Chirurg.

[61] Bialecki J, Bucsi L, Fernando N (2019). Hip and knee section, treatment, one stage exchange: proceedings of International Consensus on Orthopedic Infections. J Arthroplast.

[62] Segreti J, Nelson JA, Trenholme GM (1998). Prolonged suppressive antibiotic therapy for infected orthopedic prostheses. Clin Infect Dis.

[63] Giacometti Ceroni R, Bianchi L, Mondini A, Zagra L (2012). Peri-prosthetic infections. When to avoid surgery. Hip Int.

[64] Byren I, Bejon P, Atkins BL (2009). One hundred and twelve infected arthroplasties treated with “DAIR” (debridement, antibiotics and implant retention): antibiotic duration and outcome. J Antimicrob Chemother.

[65] Hyman JL, Salvati EA, Laurencin CT (1999). The arthroscopic drainage, irrigation, and débridement of late, acute total hip arthroplasty infections: average 6-year follow-up. J Arthroplast.

[66] Abouljoud MM, Backstein D, Battenberg A (2019). Hip and knee section, treatment, surgical technique: proceedings of International Consensus on Orthopedic Infections. J Arthroplast.

[67] Pohlig F, Mühlhofer HML, Lenze U (2017). Diagnostic accuracy of arthroscopic biopsy in periprosthetic infections of the hip. Eur J Med Res.

[68] Claassen L, Ettinger S, Pastor M-F (2016). The value of arthroscopic neosynovium biopsies to diagnose periprosthetic knee joint low-grade infection. Arch Orthop Trauma Surg.

[69] Anagnostakos Konstantinos, Meyer Christof (2019). Partial two-stage exchange at the site of periprosthetic hip joint infections. Archives of Orthopaedic and Trauma Surgery.

[70] El-Husseiny M, Haddad FS (2016). The role of highly selective implant retention in the infected hip arthroplasty. Clin Orthop Relat Res.

[71] Muñoz-Mahamud E, García S, Bori G (2011). Comparison of a low-pressure and a high-pressure pulsatile lavage during débridement for orthopaedic implant infection. Arch Orthop Trauma Surg.

[72] Urish KL, DeMuth PW, Craft DW (2014). Pulse lavage is inadequate at removal of biofilm from the surface of total knee arthroplasty materials. J Arthroplast.

[73] Hassinger SM, Harding G, Wongworawat MD (2005). High-pressure pulsatile lavage propagates bacteria into soft tissue. Clin Orthop Relat Res.

[74] Soares D, Leite P, Barreira P (2015). Antibiotic-loaded bone cement in total joint arthroplasty. Acta Orthop Belg.

[75] Tan TL, Kheir MM, Rondon AJ (2018). Determining the role and duration of the “antibiotic holiday” period in periprosthetic joint infection. J Arthroplast.

[76] Preininger B, Janz V, von Roth P (2017). Inadequacy of joint aspiration for detection of persistent periprosthetic infection during two-stage septic revision knee surgery. Orthopedics.

[77] Boelch SP, Weissenberger M, Spohn F et al (2018) Insufficient sensitivity of joint aspiration during the two-stage exchange of the hip with spacers. J Orthop Surg Res 13. 10.1186/s13018-017-0703-z 10.1186/s13018-017-0703-z PMC5763577 29321073

[78] Winkler T, Stuhlert MGW, Lieb E (2019). Outcome of short versus long interval in two-stage exchange for periprosthetic joint infection: a prospective cohort study. Arch Orthop Trauma Surg.

[79] Yusuf E, Jordan X, Clauss M (2013). High bacterial load in negative pressure wound therapy (NPWT) foams used in the treatment of chronic wounds. Wound Repair Regen.

[80] Abblitt WP, Ascione T, Bini S (2019). Hip and knee section, outcomes: proceedings of International Consensus on Orthopedic Infections. J Arthroplast.

[81] Ibrahim MS, Raja S, Khan MA, Haddad FS (2014). A multidisciplinary team approach to two-stage revision for the infected hip replacement: a minimum five-year follow-up study. Bone Joint J.

[82] Laurent E, Lemaignen A, Gras G (2019). Multidisciplinary team meeting for complex bone and joint infections diagnosis: the PHICTOS study. Rev Epidemiol Sante Publique.

[83] Ntalos D, Berger-Groch J, Rohde H (2019). Implementation of a multidisciplinary infections conference affects the treatment plan in prosthetic joint infections of the hip: a retrospective study. Arch Orthop Trauma Surg.

[84] Yan CH, Arciola CR, Soriano A (2018). Team approach: the management of infection after total knee replacement. JBJS Rev.

