# Correction to: Twenty common errors in the diagnosis and treatment of periprosthetic joint infection

**DOI:** 10.1007/s00264-019-04459-y

**Published:** 2019-12-10

**Authors:** Cheng Li, Nora Renz, Andrej Trampuz, Cristina Ojeda-Thies

**Affiliations:** 1 Charité – Universitätsmedizin Berlin, corporate member of Freie Universität Berlin, Humboldt-Universität zu Berlin, and Berlin Institute of Health, Center for Musculoskeletal Surgery (CMSC), Charitéplatz 1, 10117 Berlin, Germany; 2 Department of Traumatology and Orthopedic Surgery, Hospital Universitario 12 de Octubre, Madrid, Spain

**Correction to: International Orthopaedics**

10.1007/s00264-019-04426-7

The article **“Twenty common errors in the diagnosis and treatment of periprosthetic joint infection”**, written by Cheng Li, Nora Renz, Andrej Trampuz and Cristina Ojeda-Thies, was originally published electronically on the publisher’s internet portal (currently SpringerLink) on September 2019 without open access.

With the author(s)’ decision to opt for Open Choice the copyright of the article changed on December 2019 to © The Author(s) 2019 and the article is forthwith distributed under the terms of the Creative Commons Attribution 4.0 International License (http://creativecommons.org/licenses/by/4.0/), which permits use, duplication, adaptation, distribution and reproduction in any medium or format, as long as you give appropriate credit original author(s) and the source, provide a link to the Creative Commons licence and indicate if changes were made.

The original article was corrected.

